# Supplementary material for: Chronic Pain Prevalence and Psychosocial Burden in the Italian Population from the 2019 European Health Interview Survey
Source: Int J Environ Res Public Health. 2025 Sep 6;22(9):1395. doi: 10.3390/ijerph22091395 (PMC12469650; doi:10.3390/ijerph22091395)
Supplement: Supplementary file 1 [file ijerph-22-01395-s001.zip › ijerph-3741514-supplementary.pdf]

Table S1. Respondents and Non-Respondents characteristics				
**Respondents and Non-respondents (%) by age class and gender (not weighted sample data)**				
	**Males**		**Females**	
Age	Non-respondents	Respondents	Non-respondents	Respondents
18-24	10.3	8.5	7.2	7.1
25-34	13.5	10.9	10.0	10.1
35-44	14.7	14.3	13.0	14.0
45-54	18.1	19.5	18.4	19.0
55-64	15.9	18.0	17.9	17.2
65-74	14.6	15.2	15.1	15.2
75+	12.9	13.6	18.4	17.5
Total	100	100	100	100
**Respondents and Non-respondents (%) by age class. gender and education level (not weighted sample data)**				
	**Low**	**Medium**	**High**	**Total**
		**Males**		
Age	Non-respondents	Respondents	Non-respondents	Respondents
25-44	22.1	18.3	38.1	34.1
45-64	42.1	46.9	37.9	40.4
65+	35.8	34.7	24.0	25.5
	100	100	100	100
		**Females**		
Age	Non-respondents	Respondents	Non-respondents	Respondents
25-44	13.1	12.7	31.2	32.1
45-64	37.1	38.5	44.7	43.6
65+	49.8	48.8	24.1	24.3
	100	100	100	100

Table S2. Chronic Pain Characteristics, Causes, Treatments and Self-perceived effectiveness by sex and age classes.														
	Sex				Age								Total	
	Male		Female		18-34		35-64		65-74		75+			
	%	95% CI	%	95% CI	%	95% CI	%	95% CI	%	95% CI	%	95% CI	%	95% CI
**Pain Intensity**														
Mild	22.5	(20.9-24.1)	15.4	(14.3-16.5)	31.7	(27.2-36.1)	21.5	(20.0-23.0)	16.6	(14.5-18.7)	10.8	(9.4-12.2)	18.2	(17.3-19.1)
Moderate	52.8	(50.9-54.6)	52.2	(50.8-53.7)	49.7	(45.0-54.5)	54.4	(52.6-56.1)	54.7	(52.0-57.4)	48.5	(46.3-50.7)	52.4	(51.3-53.6)
Severe	24.8	(23.1-26.4)	32.4	(31.8-33.8)	18.6	(14.8-22.4)	24.1	(22.6-25.6)	28.7	(26.2-31.2)	40.7	(38.5-42.9)	29.4	(28.3-30.4)
**Cause**														
Surgery	7.8	(6.8-8.9)	6.7	(5.9-7.5)	3.0	(1.2-4.8)	6.6	(5.7-7.6)	8.4	(6.8-10.0)	8.0	(6.8-9.3)	7.2	(6.5-7.8)
Trauma-Injury	29.6	(27.9-31.4)	16.9	(15.7-18.1)	46.7	(41.8-51.6)	28.2	(26.6-29.8)	14.1	(12.1-16.1)	11.8	(10.3-13.3)	21.9	(20.9-22.9)
Cancer	2.7	(2.0-3.4)	3.3	(2.7-3.8)	1.8	(0.4-3.2)	2.9	(2.2-3.5)	3.4	(2.4-4.5)	3.3	(2.5-4.2)	3.0	(2.6-3.5)
Disease diagnosed	47.2	(45.3-49.1)	58.9	(57.4-60.4)	27.5	(23.1-31.9)	46.5	(44.7-48.3)	61.9	(59.2-64.6)	67.5	(65.4-69.6)	54.3	(53.2-55.4)
Disease not yet diagnosed	12.6	(11.3-13.9)	14.2	(13.1-15.3)	21.0	(16.9-25.0)	15.8	(14.5-17.2)	12.1	(10.3-14.0)	9.3	(8.0-10.7)	13.6	(12.8-14.4)
**Drugs or therapies**														
None	18.1	(16.6-19.5)	12.6	(11.6-13.6)	23.0	(18.9-27.1)	17.1	(15.8-18.5)	13.1	(11.2-15.0)	10.2	(8.8-11.6)	14.8	(13.9-15.6)
Yes, continuously	28.6	(26.9-30.4)	34.7	(33.3-36.1)	21.2	(17.2-25.2)	21.8	(20.3-23.3)	35.2	(32.6-37.9)	49.4	(47.2-51.7)	32.3	(31.2-33.4)
Yes, in cycles	15.0	(13.6-16.4)	17.1	(15.9-18.3)	14.8	(11.3-18.3)	17.3	(15.9-18.6)	17.4	(15.3-19.6)	14.4	(12.8-16.0)	16.3	(15.4-17.2)
Yes, when needed	38.3	(36.4-40.1)	35.6	(34.1-37.0)	41.0	(36.2-45.7)	43.8	(42.1-45.6)	34.2	(31.6-39.9)	26.0	(24.0-28.0)	36.6	(35.5-37.7)
**Effectiveness of treatments**														
Yes	27.0	(25.1-28.9)	21.1	(19.7-22.4)	36.2	(30.8-41.6)	27.2	(25.4-28.9)	24.2	(21.6-26.8)	14.4	(12.6-16.1)	23.3	(22.2-24.4)
Partial	64.9	(62.9-66.9)	71.7	(70.2-73.1)	54.6	(49.1-60.2)	66.2	(64.4-68.1)	70.0	(67.2-72.7)	75.8	(73.8-77.9)	69.1	(68.0-70.3)
None	8.1	(6.9-9.4)	7.3	(6.4-8.2)	9.1	(5.8-12.25)	6.6	(5.6-7.6)	5.8	(4.3-7.3)	9.8	(8.3-11.3)	7.6	(6.9-8.3)

Weighted percentages (%)
